# Treatment adherence in bipolar disorder: related factors

**DOI:** 10.1192/j.eurpsy.2025.1083

**Published:** 2025-08-26

**Authors:** F. Sahnoun, F. Cherif, D. Mnif, F. Guermazi, I. Baati, I. Feki, R. Masmoudi, J. Masmoudi

**Affiliations:** 1 Psychiatry A Department, Hedi Chaker University hospital, Sfax, Tunisia

## Abstract

**Introduction:**

It is well established that user behavior does not align with clinical recommendations regarding using medication in psychotic disorders, particularly bipolar disorder (BD). Nonadherence to treatment carries a high risk of relapse due to the recurrent nature of the illness and is influenced by multiple factors

**Objectives:**

The aim of this study is to investigate the prevalence of medication adherence among BD patients and to explore the various factors contributing to poor adherence.

**Methods:**

This cross-sectional, descriptive, and analytical study was conducted with patient diagnosed with bipolar disorder at the Psychiatry “A” Department of Hedi Chaker University Hospital. Data on clinical and sociodemographic variables were collected between March and September 2023 using a questionnaire, alongside the Medication Adherence Rating Scale (MARS) to evaluate treatment adherence.

**Results:**

A total of 37 patients with BD completed the questionnaire. The mean age was 45.4 ± 13.9 years, with a sex ratio (M/F) of 1.46.

In this study, 73 % of patients had BD type I and 27% had BD type II.

The mean MARS score was 7.14 ± 2.13 and 62.2% were adherent to their treatment.

Only 16.2% of bipolar patients discontinued their medication due to side effects, while 51.4% present unintentional poor adherence.

A significant association was found between treatment adherence and socio-economic status (p = 0.05).

The MARS score was negatively correlated with the number of hospitalizations (p = 0.05, r = -0.315)

**Conclusions:**

This study highlights that treatment adherence in bipolar disorder is influenced by various factors, with socio-economic status being a significant determinant. The negative correlation between MARS scores and the number of hospitalizations suggests that better adherence is associated with fewer hospitalizations. Improving treatment compliance is crucial to reducing the risk of recurrence and hospitalization in BD.

**Disclosure of Interest:**

None Declared

